# Adenosine triphosphatases of thermophilic archaeal double-stranded DNA viruses

**DOI:** 10.1186/2045-3701-4-37

**Published:** 2014-07-23

**Authors:** Lotta J Happonen, Susanne Erdmann, Roger A Garrett, Sarah J Butcher

**Affiliations:** 1 Department of Clinical Sciences, Division of Infection Medicine, Lund University, SE-221 84 Lund, Sweden; 2 Archaea Centre, Department of Biology, University of Copenhagen, Ole Maaløes Vej 5, DK-2200 Copenhagen N, Denmark; 3 Institute of Biotechnology, University of Helsinki, (Viikinkaari 1), P.O. Box 65, FI-00014 Helsinki, Finland

**Keywords:** ATPase, Archaeal, Virus, Genome packaging, Genome injection, MoxR ATPase

## Abstract

Adenosine triphosphatases (ATPases) of double-stranded (ds) DNA archaeal viruses are structurally related to the AAA+ hexameric helicases and translocases. These ATPases have been implicated in viral life cycle functions such as DNA entry into the host, and viral genome packaging into preformed procapsids. We summarize bioinformatical analyses of a wide range of archaeal ATPases, and review the biochemical and structural properties of those archaeal ATPases that have measurable ATPase activity. We discuss their potential roles in genome delivery into the host, virus assembly and genome packaging in comparison to hexameric helicases and packaging motors from bacteriophages.

## Introduction

Viral genomes are enclosed inside a protein capsid for protection against the environment. For many archaeal viruses, the environmental stresses may include extremes of temperature and pH, high pressure, high salt concentration and the presence of heavy metal ions. Rod-shaped viruses, such as single-stranded (ss) DNA viruses (e.g. bacteriophage M13) and ssRNA viruses (e.g. tobacco mosaic virus), usually assemble their capsid proteins as a helical tube around the genome. For such viruses, the capsid length is proportional to the length of the genome. Alternatively, some viruses can package their genomes into preformed procapsids via a specific genome packaging ATPase using energy derived from ATP hydrolysis. This is very commonly found in viruses with double-stranded (ds) DNA genomes [1]. For viruses that are not rod-shaped and do not exhibit a procapsid state, genome packaging and virus assembly mechanisms remain poorly understood. These viruses include spindle-shaped, bottle-shaped, droplet-shaped and pleomorphic viruses that are predominantly found in the archaeal domain.

This article aims to provide an overview of the different types of ATPases that are encoded by archaeal viruses. ATPases of the tailed viruses and non-tailed archaeal viruses with icosahedrally-ordered capsids are suggested to be involved in genome packaging. However, the majority of the characterised ATPases of archaeal viruses belong to viruses exhibiting different morphotypes, and these have been assigned diverse functions. We consider the functional roles of these ATPases in the life cycles of spindle-shaped archaeal viruses and in particular their inferred roles in viral genome injection into the hosts and the involvement of a MoxR-type ATPase in tail formation. Further, we highlight the genome packaging ATPase B204 of the dsDNA *Sulfolobus* turreted icosahedral virus 2 (STIV2), as it is currently the only structurally characterized archaeal viral ATPase for which the function has been determined experimentally. We focus on the ATPases of thermophilic crenarchaeal viruses because very few biochemical or structural studies have been performed on ATPases of the euryarchaea.

### Genome packaging ATPases of dsDNA viruses

Packaging of genomic nucleic acid into preformed procapsids is a complex process involving injection of the DNA (or RNA) into the procapsid via ATP or NTP-powered hydrolysis, followed by structural rearrangement of the encapsidated genome. Subsequently the DNA is transformed into a highly condensed, near crystalline state, concomitant with the maturation of the capsid. Most of our current knowledge on viral genome packaging derives from studies on dsDNA head-tail bacteriophages such as phi29 and T4, and dsRNA viruses such as phi12. First, therefore, we provide a short introduction to what is known about the conserved sequence motifs and the key components of the packaging motor proteins, together with a short summary of the well-characterized genome packaging machineries of phi29 and T4.

**Figure 1:**
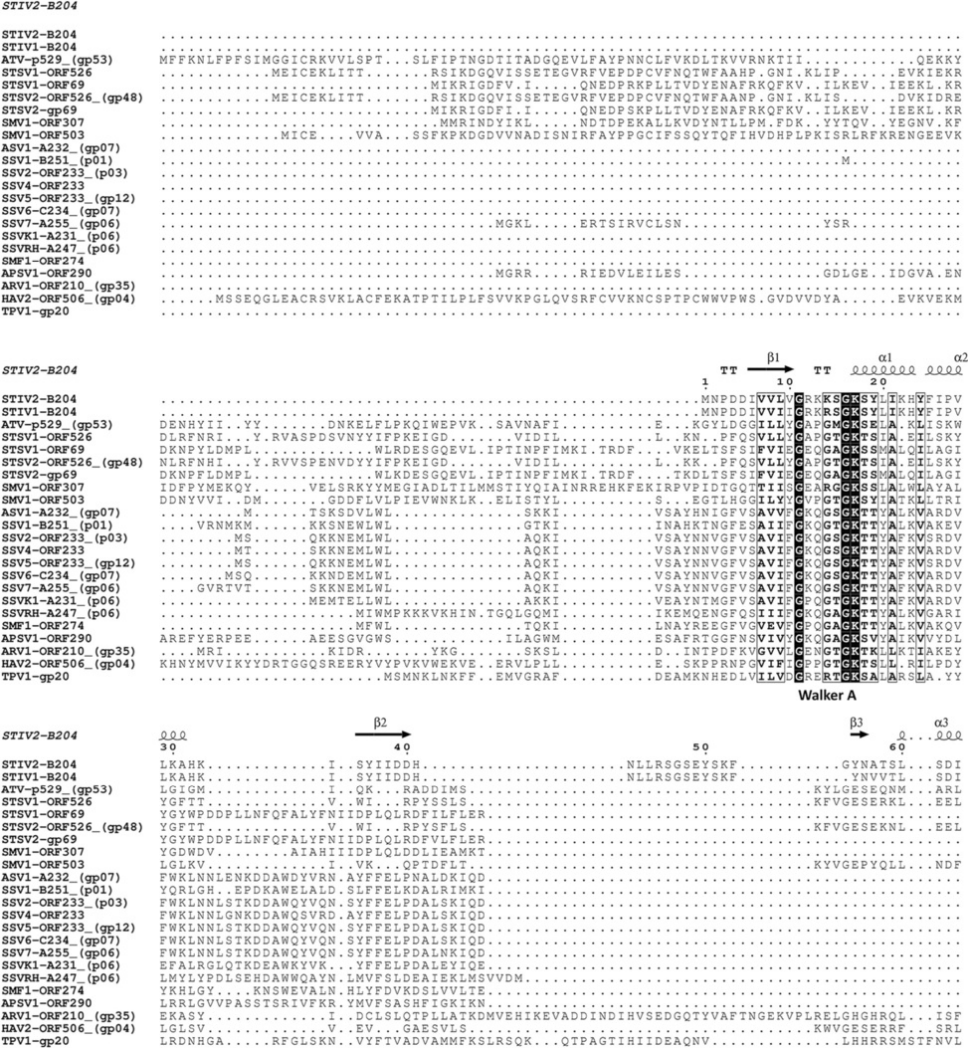
**Sequence alignment of thermophilic ATPases (part 1).** The figure shows a sequence alignment of the N-terminal regions of a selection of ATPases from Table 1 generated using Clustal Omega [16] and visualized in Espript [17]. Numbers indicate residues. The conserved Walker A motifs are labelled below the alignment. Secondary structure elements of STIV2 B204 (PDB: 4KFU) are shown above the aligned sequences.

**Figure 2:**
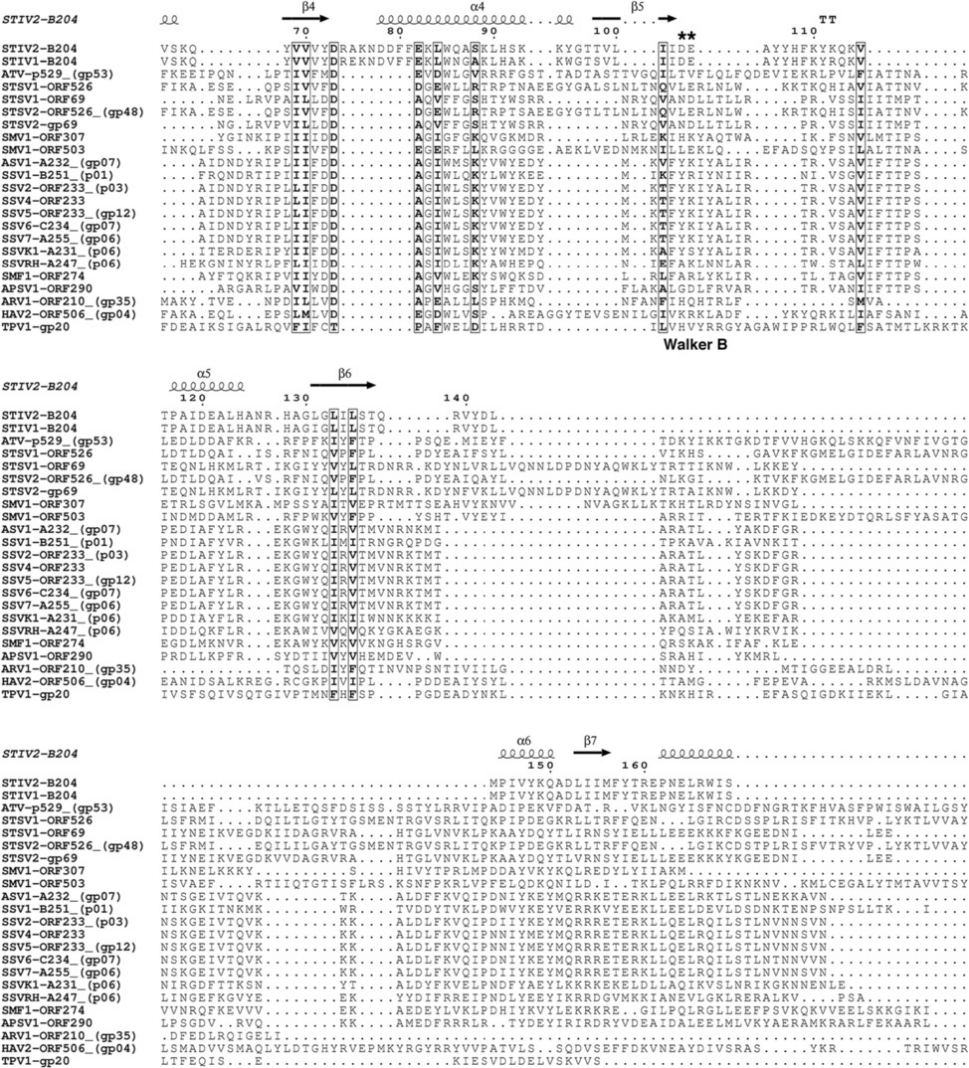
**Sequence alignment of thermophilic ATPases (part 2).** Sequence alignment of the C-terminal regions of the selection of ATPases from Table 1 as generated for Figure 1. The conserved Walker B sequence motifs are labelled below the alignment. The STIV2 B204 Walker B sequence motif is indicated with two asterisks above the alignment. Secondary structure elements of STIV2 B204 (PDB: 4KFU) are shown above the aligned sequences.

A large variety of genome packaging ATPases have been characterised and they exhibit highly conserved sequence motifs at their active sites (Figures 1 and 2), which include the phosphate-binding loop (P-loop or Walker A sequence motif) and the Walker B sequence motif [2-4]. The consensus sequences of these motifs are GXXXXGK(T/S) and hhhhDE, respectively, where X denotes any amino acid and h indicates any hydrophobic amino acid [2,4]. The conserved lysine in the Walker A motif participates in nucleotide binding and the conserved glutamate in the Walker B motif facilitates activation of a water molecule for the hydrolysis reaction [5]. The Mg^2+^-ion required for ATP hydrolysis can be coordinated either by the conserved aspartate in the Walker B domain [2,4,6] or by the conserved serine in the Walker A domain [7-9]. Arginine fingers, located either on the same subunit as the Walker A and B motifs, or on an adjacent subunit, facilitate formation of the transition state, and they are inserted into the catalytic site as a result of a conformational change that occurs prior to the catalytic step [10-12]. Two additional sequence motifs - the sensor 1 and 2 regions - are conserved in AAA+ family proteins. These regions are considered to detect the status of the bound nucleotide, distinguishing between ATP and ADP [13], interacting with the γ-phosphate of the ATP [14,15].

Tailed dsDNA bacteriophages contain two key components for genome packaging: the ring-forming portal protein (the connector) through which DNA is translocated, and the translocating motor (the terminase or ATPase). The terminase is usually hetero-oligomeric with a small subunit involved in recognition of the incoming DNA and a large subunit containing the ATPase domain and a structural motif for docking at the portal vertex. In some ATPases, an endonuclease activity is also present which cuts concatemers of the genome upon packaging (as reviewed by [1,18]).

### The phi29 genome packaging machinery

The genome packaging machinery of the podovirus phi29 is one of the best-studied, most powerful nanomotors known. Using optical tweezers it has been shown to package DNA at an initial rate of 100 bp s^−1^ against an average pressure of 57 pN [19]. The phi29 packaging motor consists of the dodecameric connector protein (gp10) [20], the hexameric ATPase (gp16) containing the canonical Walker A and B sequence motifs [21,22] and the phage-encoded hexameric packaging RNA (pRNA) ring. The packaging mechanism of this motor has recently been reviewed [23]. Current findings show that the hexameric gp16 motor protein functions by revolution rather than rotation [24]. For gp16-driven translocation to occur, one hexamer subunit binds ATP which stimulates a change to a conformation susceptible to DNA binding. Hydrolysis of the bound ATP produces a second conformational change which results in displacement of the DNA away to an adjacent ATP-bound subunit. A single ATP molecule is hydrolysed in each transitional step, and a total of six ATP molecules are consumed for one helical turn of 360°. The energy released from hydrolysis of each ATP propels the DNA forward by 1.75 bp [24].

### The T4 genome packaging machinery

The bacteriophage T4 packaging motor consists of the dodecameric connector (gp20) [25], the pentameric large terminase motor (gp17) [26] and the 11- or 12-meric small terminase regulatory protein (gp16) [27]. Similarly to the phi29 gp16 protein, the T4 gp17 motor protein has been shown to generate a force of 60 pN [28]. However, the average speed produced by gp17 is much higher than that of phi29 gp16 – 700 bp s^−1^ – and it can reach velocities up to 2000 bp s^−1^[28]. The gp17 protein carries an N-terminal ATPase domain, and a C-terminal endonuclease domain to cleave the genome upon headful packaging [12,29]. The two domains are linked by a hinge region which enables the domains to move between “tensed” and “relaxed” states, thereby translocating the T4 genome in a “piston-like” fashion [12,26]. Both the genomic DNA to be packaged and the ATP molecule used as fuel, bind to the same T4 gp17 subunit which induces a conformational change leading to the translocation event [12,26,30]. At any time during the packaging event, one subunit of gp17 is in the “tensed” state, and the others are in the “relaxed” state [26]. Energy released by hydrolysing a single ATP molecule translocates the DNA by two base pairs into the procapsid – an amount very similar to that of the phi29 gp16 packaging ATPase [26,30].

Essential questions regarding the mechanism of genome packaging in tailed dsDNA viruses have been addressed by Schwartz et al. [30,31] and Sun et al. [12,26]. Moreover, studies on phi12 P4 have provided insights into how this might work in dsRNA viruses [10,32,33]. However, the genome packaging mechanisms of icosahedrally-ordered dsDNA viruses lacking a connector or tail are poorly characterized. Current understanding is based primarily on what is known about the genome packaging ATPase P9 of bacteriophage PRD1 which stems from genetic studies and in vitro experiments using cell lysates. The ATPase itself has been intractable to structural characterization [34,35]. In PRD1-like viruses, with an ordered icosahedral capsid and an internal lipid membrane, small membrane proteins (P20 and P22 in PRD1) have been inferred to form a membrane pore at the packaging vertex, and they may function similarly to the connectors of head-tail phages [36]. The packaging rate of the PRD1 machinery is 340 bp s^−1^ based on the time of the appearance of the first infectious particles [35], a value close to that described for phi29 gp16 and T4 gp17. We have recently gained some insight into the structure of a genome packaging ATPase from a PRD1-like virus, with the newly solved crystal structure of the STIV2 genome packaging ATPase, B204 [7] (discussed below).

### ATPases of archaeal double-stranded DNA viruses

#### Predicted roles of thermophilic archaeal viral ATPases

Although this review is focused mainly on ATPases involved in archaeal viral genome packaging, ATPases also perform other important and diverse functions in viral life cycles. Nucleoside triphosphate (NTP)-hydrolysing enzymes are encoded in many of the archaeal viral genomes so far described (Table 1, Figures 1 and 2). The seven major functions associated with these ATPases are moving viral DNA into the host (DNA entry), packaging viral genomes into a preformed viral capsid, initiating DNA replication, repairing DNA, contributing to protease activity (Lon protease), acting as chaperones in protein folding (MoxR ATPases), and working as transporters (Table 1). These functional predictions rely to a large extent on sequence similarity searches performed using the tools BLAST and HHpred rather than on experimental evidence. For multidomain proteins we have tended to use the alignment of the ATPase domain as the determinant of function.

**Table 1:** Functions of archaeal thermophilic viral ATPases

**Crenarchaeal**	**Virus**	**ORF**	**NCBI/ENA reference sequence**	**Postulated function**	**Reference**
***Sulfolobus*** **turreted icosahedral virus**	STIV	*b204*	YP_025021.1	DNA packaging	[37]
	STIV2	*b204*	YP_003591106.1	DNA packaging	[7]
**Spherical**	PSV	ORF582 (gp02)	YP_015523.1	DNA packaging	[38]
	TTSV1	ORF1 (gp01)	YP_164342.1	DNA packaging	[39]
***Bicaudaviridae***	ATV	p529 (gp53)	YP_319884.1	DNA entry	[40]
		p618 (gp66)	YP_319897.1	MoxR-type chaperone	[41]
**Monocaudaviruses**	STSV1	ORF526 (gp69)	YP_077262.1	DNA repair	[42]
	STSV2	ORF526 (gp48)	YP_007348292.1	DNA repair	[43]
	SMV1	ORF503	CDF81345.1	DNA entry	[44]
		ORF307	CDF81374.1	DNA repair	
		ORF588	CDF81351.1	MoxR-type chaperone	
***Fuselloviridae***	ASV1	*a232* (gp07)	YP_003331412.1	Lon protease	[45]
	SSV1	*b251* (p01)	NP_039777.1	Lon protease	[46]
	SSV2	ORF233 (p03)	NP_944455.1	Lon protease	[47]
	SSV4	ORF233	YP_001552190.1	Lon protease	[45]
	SSV5	ORF233 (gp12)	YP_002221477.1	Lon protease	[48]
	SSV6	*c234* (gp07)	YP_003331457.1	Lon protease	[45]
	SSV7	*a255* (gp06)	YP_003331489.1	Lon protease	[45]
	SSVK1	*a231* (p06)	NP_963972.1	Lon protease	[49]
	SSVRH	*a247* (p06)	NP_963931.1	Lon protease	[50]
	SMF1	ORF274	YP_007678010.1	Lon protease	[51]
	APSV1	ORF290	CCD22100.1	Lon protease	[52]
***Lipothrixviridae***	AFV1	ORF426	YP_003740.1	Lon protease	[53]
	AFV2	ORF425 (gp15)	YP_001496940.1	Lon protease	[54]
***Rudiviridae***	ARV1	ORF210 (gp35)	YP_001542652.1	ABC transporter	[55]
		ORF443 (gp16)	YP_001542633.1	Lon protease	
	SIRV1	ORF440 (gp11)	NP_666599.1	Lon protease	[56]
	SIRV2	ORF436 (gp18)	NP_666552.1	Lon protease	[56]
	SRV	ORF440	CAQ58449.1	Lon protease	[57]
	SMR1	ORF439 (gp08)	YP_006990086.1	Lon protease	[58]
**Tadpole-shaped**	HAV2	ORF506 (gp04)	YP_003773387	DNA repair	[59]
**Euryarchaeal**					
	TPV1	gp02	YP_005271224.1	DNA replication	[60]
		gp20	YP_005271242.1	ABC transporter	
		ORF560	AEY69051.1	DNA replication	

Thermophilic archaeal viruses for which no ATPase domains were detected include the following with genome accession numbers <http://www.ebi.ac.uk/genomes/archaealvirus.html>. Ampullavirus ABV (EF432053), lipothrixviruses SIFV (AF440571), AFV3 (AM087121), AFV6 (AM087121), AFV7 (AM087122), AFV8 (AM087123), AFV9 (EU545650), *Thermoproteus tenax* virus TTV1 (X14855), Hyperthermophilic archaeal virus HAV1 (GU722196), *Aeropyrum* spring-shaped virus (HE681887), *Aeropyrum pernix* oxoid virus (HE580237) and Archaeal BJ1 virus (AM419438).

Despite the diversity of their suggested functions, many of the currently annotated thermophilic viral ATPases align well over the Walker A sequence motif (Figures 1 and 2). These ATPases are found within the genomes of the crenarchaeal viruses belonging to the *Bicaudaviridae* (*Acidianus* two-tailed virus [ATV]); Monocaudaviruses (*Sulfolobus tengchongensis* spindle-shaped Virus [STSV1, STSV2], and *Sulfolobus* Monocauda Virus 1 [SMV1]); *Fuselloviridae* (*Sulfolobus* spindle-shape virus [SSV1-7, SSVRH, SSVK1, SMF1]; *Acidianus* spindle-shaped virus 1 [ASV1] and *Aeropyrum pernix* spindle-shaped virus 1 [APSV1]); *Rudiviridae* (*Sulfolobus islandicus* rod-shaped viruses [SIRV1, SIRV2], Sulfolobales Mexican rudivirus 1 [SMR1], *Acidianus* rod-shaped virus 1 [ARV1] and *Stygiolobus* rod-shaped virus [SRV]); *Lipothrixviridae* (*Acidianus* filamentous viruses [AFV1 and AFV2]); tadpole-shaped hyperthermophilic archaeal virus 2 (HAV2), and the *Sulfolobus* turreted icosahedral viruses (STIV, STIV2) (Table 1). Interestingly, all but one of the currently annotated ATPases encoded by the *Rudiviridae* and *Lipothrixviridae* genomes are predicted to function as Lon proteases.

Six annotated ATPases do not align with the other groups (Table 1). They include the predicted genome packaging ATPases of the spherical viruses *Pyrobaculum* spherical virus (PSV) and *Thermoproteus tenax* spherical virus 1 (TTSV1), the MoxR ATPases, p618 of ATV and ORF588 of SMV1, and the ATPases of *Thermococcus prieurii* virus 1 (TPV1) gp20 and ORF560 (Table 1). While most thermophilic archaeal viruses carry one ATPase encoding gene on their genome, some have two (STSV1, STSV2, ARV1 and ATV) or even three (SMV1 and TPV1) (Table 1). For the latter groups, the ATPases are predicted to exhibit different functions, except for TPV1 where two ATPases are predicted to participate in DNA replication.

All of the current structural and biochemical data on ATPases of archaeal viruses stem from studies of thermophilic archaeal viruses and little is known about euryarchaeal viral ATPases of the haloarchaea or methanogens. Three thermophilic viral ATPases that have been studied in some detail are discussed in this review: one involved in DNA packaging (STIV2 B204) [7], another implicated in DNA entry into the host (ATV p529) [40], and a third operating as a MoxR-type chaperone protein (ATV p618) [41].

### ATPases of haloarchaeal viruses

Although several haloarchaeal viruses are predicted to carry packaging ATPases, the enzymes are poorly characterized experimentally. The predictions are based primarily on the presence of the canonical Walker A and B sequence motifs and, for viruses with icosahedrally-ordered capsids and internal lipid membranes, packaging ATPases have been predicted for SH1 (ORF17) [61], SNJ1 (gene 23) [62] and SSIP-1 (gp40) [63]. ATPases have also been predicted in the head-tail viruses HVTV-1 (ORF59) and HSTV-2 (ORF52) [64] and HSTV-1 (ORF1) [65]. In addition, putative ATPases have been described for viruses without an icosahedrally-ordered capsid and a postulated procapsid state, including the pleolipovirus His2 (ORF 33) [66,67] and the spindle-shaped His1 (ORF 16) [68]. The precise roles of these ATPases in the viral life-cycle, for instance, whether or not they participate in genome packaging, remain to be determined.

### Biochemical properties of thermophilic viral ATPases

#### ATPases of the ATV bicaudavirus

The bicaudavirus ATV was isolated from a hot spring at the Solfatara solfataric field in Pozzuoli (Italy) with a temperature range of 87 to 93°C, and a pH range of 1.5 to 2 [69]. The virus particle is spindle-shaped, with a tail at each end; hence the name. The life cycle of ATV is exceptional with the tail development being extracellular and independent of its host [69,70]. The circular dsDNA ATV genome is 62 kb, encoding 72 putative proteins, of which at least 11 are found within the virion. Currently two ATPases, p529 and p618, have been characterised experimentally [40,41]. ATV p529 was inferred to facilitate viral genome injection into the host, whereas p618 was demonstrated to be a MoxR-type ATPase and was assigned a chaperone role in extracellular tail development (Table 1) [40,41]. p529 homologs occur in the other large single-tailed fusiform viruses STSV1 (ORF526), STSV2 (ORF526 [gp48]) and SMV1 (ORF503) (Table 1), while the MoxR AAA+ ATPase p618 is present in ATV virions with a potential homolog encoded by SMV1 (ORF588) (Table 1).

**Figure 3:**
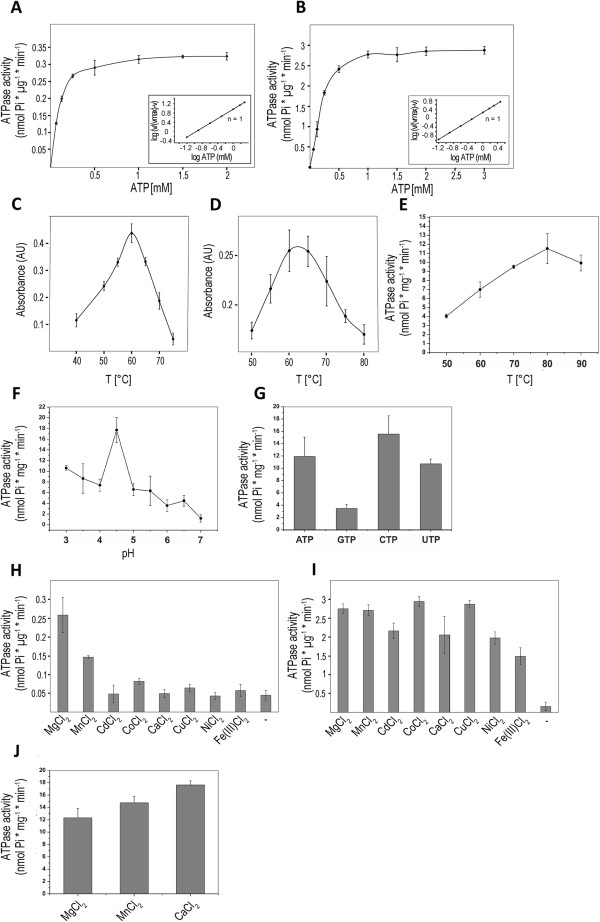
**Biochemical properties of thermophilic viral ATPases.** The release of inorganic phosphate (Pi) during nucleotide hydrolysis by ATV p529 and p618 and STIV2 B204 was determined by the malachite green assay. The ATPase activity of B204 is reported as nmol × min^−1^ × mg^−1^, and that of p529 and p618 as nmol × min^−1^ × μg^−1^. **(A)** 0.015 mg/ml of p529 and **(B)** 0.01 mg/ml of p618 were incubated at 60°C with increasing amounts of ATP. K_m_ values of 0.11 mM for p529, and 0.55 mM for p618 were determined. The maximum ATP hydrolysis velocity was 0.57 nmol × min^−1^ × μg^−1^ and 4.55 nmol × min^−1^ × μg^−1^ for p529 and p618, respectively. The inset shows a Hill plot, with a Hill coefficient of n = 1. The temperature dependence of the ATPase activity was analysed in the presence of 1 mM ATP for p529 **(C)** and p618 **(D)**, and in the presence of 2 mM ATP at pH 4.5 for B204 **(E)**. **(F)** The influence of pH on the activity of B204 ATP hydrolysis at 80°C. **(G)** Nucleotides hydrolysed by B204 at pH 4.5 80°C. Nucleotide hydrolysis depends on the presence of divalent cations. ATV p529 preferentially utilizes Mg^2+^**(H)**, while p618 **(I)** and B204 **(J)** are less specific. A low background concentration of inorganic phosphate in panels **H** and **I** originates from a contamination of the protein preparation. The figure has been modified with permission from [7,40,41]. Copyright© American Society for Microbiology.

p529 and p618 resemble some genome packaging ATPases of tailed bacteriophages, including T4 gp17, in that they carry two separate domains; an N-terminal domain with ATPase activity and a C-terminal domain associated with different functions; the C-terminal domain of p529 binds to DNA. p529 and p618 hydrolyse ATP [40,41]. The Km value of p529-driven ATP hydrolysis is 0.11 mM and that of p618 0.55 mM, and maximum velocities are 0.57 nmol × min^−1^ × μg^−1^ and 4.55 nmol × min^−1^ × μg^−1^, respectively (Figure 3A and B) [40,41]. Optimal reaction temperatures determined in vitro were 65°C for p529 and 60°C for p618 (Figure 3C and D), significantly lower than the optimal growth temperature (85°C) of the viral host [40,41]. ATV p529 exhibits a high specificity for Mg^2+^ as a cofactor, whereas p618 shows lower specificity, although both enzymes are active in the presence of a wide range of different divalent cations (Figure 3H and I) [40,41]. Since p618 is likely to be active within the virion with limited access to Mg^2+^, utilization of alternative cofactors may be important for its function.

Both p529 and p618 oligomerize upon ATP binding, forming double (p529) or single (p618) hexameric rings [40,41]. In contrast to p618, which readily forms stable oligomers, p529 is only able to oligomerize in vitro when the Walker B motif glutamate is mutated (E177Q), suggesting that dissociation of the complex occurs after ATP hydrolysis. Oligomerization and ATPase activity of the isolated ATPase domain (amino acids 1–315) were only observed for p529 [40].

### The genome packaging ATPase B204 of the icosahedral STIV2

STIV2 infects *Sulfolobus islandicus* and is an icosahedrally-symmetric virus with an internal membrane and vertices decorated by large turrets thought to be involved in host-cell recognition and attachment [71]. It was isolated from an acidic hot spring (88.3°C, pH 3.5), and has a circular 16.6 kb dsDNA genome encoding 34 predicted proteins, nine of which encode structural proteins [7,71]. An STIV2-encoded ATPase – B204 was predicted on the basis of sequence similarity to other P-loop ATPases [71]. This ATPase is most active at pH 4.5 and 80°C, close to the optimal physiological conditions of the host [7,71] (Figure 3E and F). The temperature optimum of B204 is 20°C to 25°C higher than that of ATV p618 and p529 and B204 can also hydrolyse GTP, UTP, and CTP [7] (Figure 3G). Moreover, as shown for ATV p618, B204 can also utilize other cofactors in addition to Mg^2+^[7] (Figure 3J).

In contrast to ATV p529, B204 does not separate NTP hydrolysis and DNA binding into distinct domains. It has been demonstrated via electrophoretic mobility shift assays that B204 binds both ds and ss, linear and circular DNA, with no apparent sequence specificity, but linear dsDNA stimulated ATPase activity [7]. In this respect it resembles the phi29 genome packaging ATPase gp16, which also binds DNA in a non-specific manner, and its ATPase activity is stimulated by DNA [21,72]. The minimal length of a B204-bound DNA was estimated at 20 bp [7], similar to the minimum length of a DNA molecule bound to T4 gp17 [73]. However, B204 also binds RNA in addition to DNA [7].

### Structural properties of thermophilic viral ATPases

**Figure 4:**
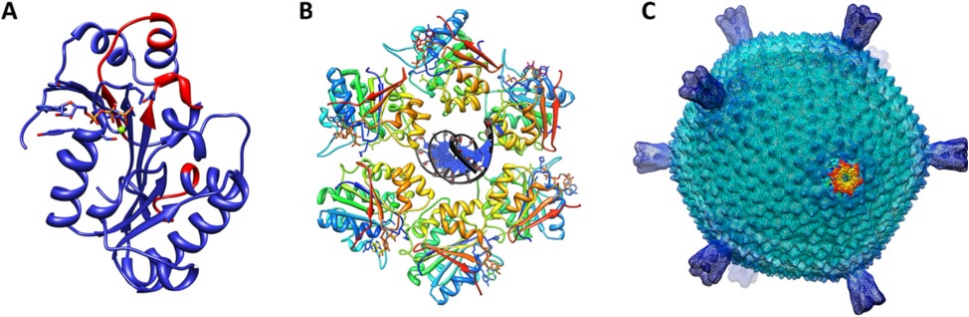
**B204 structure. (A)** Ribbon diagram of a B204 monomer bound to AMPPCP and Mg^2+^ (green sphere) (PBD: 4KFU) [7]. The regions highlighted in red indicate the conserved P9-motif described for the PRD1-like lineage of viruses [34]. **(B)** A hexamer model of B204 shown in rainbow hues (blue to red) generated by aligning six monomers of chain A of the AMPPCP B204 structure (PDB: 4KFU) onto each of the FtsK monomers in the FtsK hexamer (PDB: 2IUU) using the program UCSF Chimera [76]. According to our hypothesis the C-terminal part of B204 (orange to red) lies on the capsid distal side. A short stretch of dsDNA (black and blue helix) is modelled into the channel formed by the monomers. **(C)** A radially depth-cued isosurface representation (at 2 σ above the mean) of the 20-Å resolution reconstruction of STIV2 (EMDB: 1679) [71] with one of the turrets replaced by our hexameric model of B204 (surface representation in rainbow hues as in panel B). The figure was rendered in UCSF Chimera [76].

NTPase B204 is currently the only thermophilic viral dsDNA packaging NTPase for which the structure has been determined. B204 is a 24.8 kDa monomer exhibiting nine β-strands and eight α-helices organised in the topology of the FtsK-HerA superfamily (Figure 4A) [3,6,7]. The sequence shows similarity to that of genome packaging ATPases of membrane-containing dsDNA viruses of the PRD1-like family [34,74,75].

**Figure 5:**
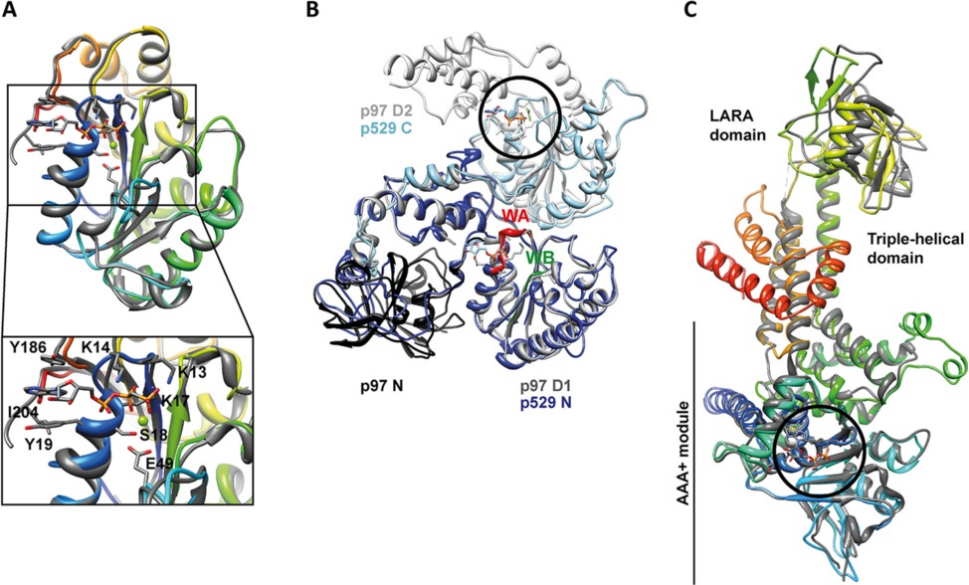
**Homology modelling of STIV B204, ATV p529 and ATV p618.** Reliable homology models for STIV B204, ATV p529 and ATV p618 were generated using the I-TASSER server [77]. **(A)** STIV B204 (rainbow) and STIV2 B204 (PDB: 4KFU) (grey). The residues that interact with the ligand are shown for STIV2 B204 (inset). **(B)** ATV p529 (shades of blue) and the AAA+ ATPase p97 (PDB: 3CF1) (shades of black and grey). The p97 domains [78] are indicated (N: black; D1: dark grey; D2: light grey). The p529 ATPase active N-terminal domain (dark blue) maps to the p97 N- and D1 –domains, and the p529 C-terminal endonuclease active domain (light blue) to the p97 D2-domain. The p97 nucleotide binding site in the D2-domain is highlighted (black circle), and the p529 Walker A and B domains are indicated (red and green, respectively) mapping onto the nucleotide binding site of p97 domain D1. **(C)** ATV p618 (rainbow) and the AAA+ ATPase RavA (PDB: 3NBX) (grey) [79]. The domain organisation of RavA is indicated, and the active site is highlighted (black circle). The figure was rendered in UCSF Chimera [76].

Based on the atomic models, the residues interacting with the hydrolysable NTP are the Walker A motif residues K13, K14, G16, K17 and S18, and residues Y19, Y186 and I204 (Figure 5A) [7]. The nucleoside moiety stacks between Y19 and Y186, but the sugar moiety does not interact with B204 [7]. The catalytic Mg^2+^-ion is coordinated by the Walker A motif residue S18 [7]. Two active site conformations were observed depending on the presence or absence of a nucleotide in the P-loop [7]. The active site pockets lacking a nucleotide are in the closed conformation, whereas the active site pockets occupied by a nucleotide are in the open conformation [7]. The maximum difference between these two conformers is 1.8 Å [7]. The major differences between the closed and open conformers are as follows: in the absence of a nucleotide, i) the gap between Y19 and Y186 opens up; ii) K13 and K14 bend away from the nucleotide binding site; iii) E49 – the proposed glutamate responsible for the activation of a water molecule for the hydrolysis reaction – moves away from the active site, and iv) S18 shifts away from the Mg^2+^-ion binding site. Notably, in B204 the conserved glutamate in the Walker B motif (E104) that is considered to be responsible for water activation in many other ATPases [5] lies too far from the active site to participate in catalysis.

The X-ray structures of B204 (PDB: 4KFR-4KFU) yielded a theoretical model for the catalytic cycle of the hexameric enzyme. The catalytic Mg^2+^-ion requires the presence of at least one nucleotide diphosphate and, in the absence of a β-phosphate, the S18 side chain is displaced and the catalytic metal site does not form [7]. This suggests that the metal ion arrives with the NTP and leaves with the NDP product [7]. It was proposed that at least two adjacent active sites, on neighbouring subunits, are required for hydrolysis. On nucleotide binding, the active site pocket opens up and this opening is most likely transmitted by a hinge-like movement of the B204 helix α1 around the α1-β2 loop. Simultaneously, the space between Y19 and Y186 closes around the nucleoside moiety and the active site residues K13 and K14 bend down to make contact with the nucleotide being hydrolysed. The E49 glutamate responsible for activating the water molecule that is required for hydrolysis moves closer to the active site. The proposed arginine finger R127, from a neighbouring subunit would, as a result of a conformational change, reach into the active site of the hexamer during hydrolysis and stabilize the transition state. Although the force promoting this conformational breathing of the monomers remains to be elucidated, we have identified two loop-regions (loops β2-β3 and β4-α3) located in the interface between two subunits that when subjected to sequential Zn^2+^-ion binding and release could, possibly, induce these changes [7].

It is difficult to envisage how the STIV2 genome translocation by B204 could be similar to that of T4 gp17 because B204 does not exhibit two separate domains that could move relative to one another [7,12,26]. Moreover, although tail-less STIV2 lacks key components of the phi29 genome packaging system, including the connector and pRNA, a similar genome packaging mechanism by revolution rather than rotation could take place. In viruses such as the PRD1-like viruses (including STIV2), with an ordered icosahedral capsid and an internal lipid membrane, small membrane proteins have been suggested to generate a pore in the membrane at the packaging vertex and they may function like the connectors of head-tail phages [80]. There are several such proteins encoded by STIV2 that could fulfil this function [7,71]. Possibly the translocation mechanism of B204 is similar to that of phi29 gp16 in that one ATP molecule is first bound to one subunit of the proposed hexameric structure. This would lead to a conformational change rendering B204 susceptible to DNA binding. Subsequent ATP hydrolysis-induced conformational changes, possibly aided by the B204 loops β2-β3 and β4-α3, might then shift the DNA molecule to the adjacent ATP bound subunit. The current drawbacks to this model are: i) STIV2 lacks some key components of the phi29 packaging system and ii) B204 binds DNA in the absence of ATP under our assay conditions [7],The exact role of each of the residues in the Walker A and B motifs in the catalysis remains to be elucidated although it is clear that residue S18 coordinates the metal ion. Mutagenesis of K17 in the Walker A domain, and of E104 in the Walker B motif, into alanine residues did not affect B204 ATP hydrolysis and, therefore, they are not essential for hydrolysis by monomeric B204 (L.J.H., unpublished). Attempts at producing a biological hexamer of STIV2 B204 have so far been unsuccessful and our conclusions are based on a hexameric model (Figure 4B and C). An active hexameric form of B204 would, in combination with mutagenesis studies, provide valuable insights into the distinct roles of each residue during the catalytic cycle.

### Homology modelling of the thermophilic viral ATPases

The closest known relative to STIV2 is STIV [71,81]. Originally, the predicted STIV genome packaging ATPase was described as a 164 amino acid protein, but it was later confirmed that the original annotation was too short due to a frame-shift sequencing error (M. Young, personal communication). We predicted a revised gene product for the STIV ATPase, with a length of 204 amino acids, and an amino acid sequence identity of 90.2% to STIV2 B204. Therefore, we modelled the STIV B204 using I-TASSER (Table 2) [77] in order to explore the tertiary structure conservation between these two thermophilic ATPases (Figure 5A). The RMSD (root-mean square deviation) between our B204 structure complexed with AMPPCP (PDB: 4KFU) and the STIV B204 model is 2.5 ± 1.9 Å (Table 2), with well-conserved active site topology (Figure 5A, inset). Based on the high sequence identity and the apparent conserved active site topology, we propose that the STIV B204 residues interacting with the ATP are conserved between the two viruses (even though STIV2 B204 K14 is replaced by R14 in STIV) (Figure 1). Furthermore, it is highly likely that the catalytic cycles of the two ATPases are similar.

**Table 2:** Homology modelling of thermophilic viral ATPases

**Protein**	**Length (AA)**	TM-score [0, 1]*	**RMSD (Å)**	**Modelled amino acids**	**Main parent model (PDB id)**	**Reference**
STIV B204	204	0.92 ± 0.06	2.5 ± 1.9	Met_1_-Ile_204_	4KFU**	[7]
ATV P529	529	0.61 ± 0.14	9.4 ± 4.6	Met_1_-Tyr_529_	3CF1 (p97)	[78]
ATV P618	618	0.57 ± 0.15	10.6 ± 4.6	Met_1_-Ser_618_	3NBX (RavA)	[79]

*A TM value > 0.5 implies that the model has the correct topology [82].

**The STIV2 B204 structure in complex with AMPPCP (PDB: 4KFU) was used as a user-defined restraint.

We also generated homology models for ATV p529 [40] and p618 [41] using I-TASSER [77] (Table 2) in order to gain insight into their possible structural and functional conservation. They were inferred to be homologous with the AAA+ ATPases p97 and MoxR, respectively [40,41]. The main parent model for p529 used in our homology modelling with I-TASSER was the hexameric type II AAA+ ATPase p97 containing two nucleotide-binding domains D1 and D2, where the former is inactive under physiological conditions [78,83] (Figure 5B). Two distinct domains were described for ATV p529: the N-terminal domain carrying ATPase activity (Figure 5B, dark blue), and the C-terminal domain exhibiting endonuclease activity (Figure 5B, light blue) [40]. This correlates with descriptions of terminase-proteins of head-tail viruses, with one domain for DNA-binding and another with ATPase activity [1,18]. In our model, the p529 N-terminal ATPase active site maps on top of the p97 D1 nucleotide binding site (Figure 5B).

The main parent model for p618 used in our homology modelling was the hexameric RavA MoxR AAA+ ATPase [79]. RavA consists of three domains: i) the N-terminal AAA+ module (residues 1–306); ii) the triple-helical domain (residues 307–330 and 442–497); and the iii) LARA domain [79]. The overall topology of p618 follows that of RavA, except for the three C-terminal helices present only in p618 (orange to red helices in Figure 5C). The overall topology at the active site is highly conserved for both proteins.

### Role in virus life cycle and relationship to other viruses

**Figure 6:**
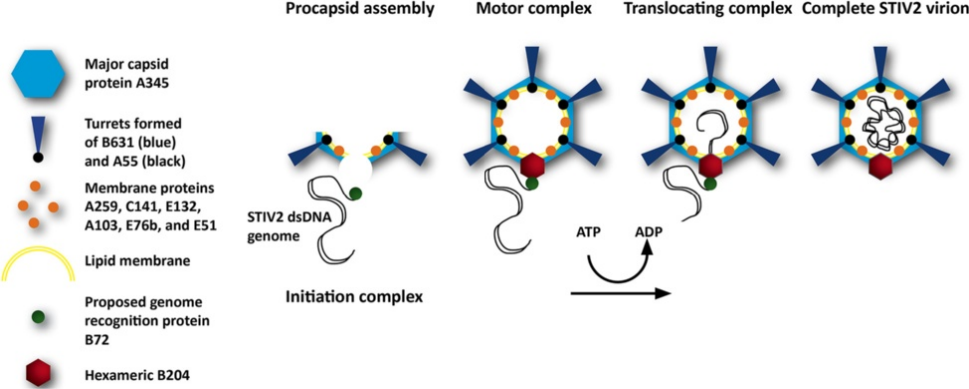
**Model for STIV2 genome packaging.** Schematic model for genome encapsidation of STIV2. The capsid of STIV2 is composed of A345, the major capsid protein (light blue hexagon). The host-attachment structures – the turrets – are composed of B631 (dark blue triangles) and the membrane protein A55 (black spheres), as described for STIV [81]. The capsid encloses the viral lipid membrane (yellow), with several predicted membrane proteins (orange spheres) attached to it. The membrane proteins E51 and E76b are proposed to form a pore in the membrane for STIV2 genome translocation at the packaging vertex [7]. STIV2 assembly initiates by the formation of the procapsid. We have proposed that the postulated STIV2 genome recognition protein B72 (green sphere) would simultaneously recognize and bind to the STIV2 genome, forming the initiation complex thus recruiting the viral genome for packaging [7]. According to our hypothesis, the initiation complex is recognised by the genome packaging ATPase B204 that has assembled as a hexamer (red) at the packaging vertex. The STIV2 genome is translocated into the procapsid via ATP powered hydrolysis. The figure is redrawn from [7].

Viruses with icosahedrally-ordered capsids package their genomes into a preformed procapsid with the help of a translocating ATPase. One such ATPase is STIV2 B204 and, based on our biochemical and structural data combined with the knowledge of the assembly of other membrane-containing and non-tailed viruses such as STIV and PRD1, we have proposed a model for the role of B204 in STIV2 (and STIV) assembly (Figure 6A) [7]. B204 binds DNA unspecifically [7] which suggests that it does not bind to a specific packaging sequence on the STIV2 genome. This led us to the proposal that another STIV2 DNA-binding protein is responsible for the recognition and recruitment of the genome to the putative packaging vertex. Currently a single DNA-binding protein B27 has been detected in the virion [71] and other possible co-functional DNA-binding proteins could be STIV2 B116 or F98, for which the STIV counterparts B116 and F93 have been shown to bind DNA but their functional roles remain unclear [84,85]. According to our model, procapsid assembly would begin at the postulated packaging vertex concurrently with the STIV2 genome being recognized by B72. Small membrane proteins, most likely E51 and E76b, would form a pore in the viral membrane for genome translocation. Recruitment of STIV2 DNA by B72 to the packaging vertex would generate the active motor complex that, on ATP hydrolysis, would translocate the DNA into the procapsid. We propose further that the STIV2 DNA is packaged as a linear molecule which is circularized within the capsid although no ligase gene has yet been identified. STIV B204 remains associated with the virion and is not dissociating after packaging [86].

### ATV p529 might be involved in genome injection

ATV p529 has been proposed to participate in viral DNA delivery into the host [40]. To date, most of our knowledge on viral DNA injection into the host and its subsequent release is derived from studies on tailed bacteriophages, although a few archaeal viral studies have been performed [87]. Viral DNA is packaged at a very high density within virions, and several in vitro studies have shown that this pressurized DNA can provide the force for DNA ejection [88]. In contrast, DNA injection has been shown to be an active process involving both push and pull forces that are mediated by viral and host proteins and may require ATP. For example, phi29 DNA is injected via a two-step “push-pull” mechanism. Initially about 65% of the phi29 DNA in pushed into the host cell, probably as a result of the pressure inside the viral capsid. Subsequently the remaining DNA is pulled into the host cell facilitated by viral protein gp17 in an energy-dependent process, although gp17 has not been associated with ATPase activity [89,90]. To date, DNA ejection from archaeal virions has received little attention. One of the few viruses that has been studied is the spindle-shaped haloarchaeal virus His1 [87] and, as for phi29, it was shown that His1 DNA ejection is only partial, and most likely requires host factors for completion [87]. Moreover, apart from ATV p529 described here, we are not aware of other archaeal ATPases postulated to participate in viral DNA entry into a host.

To date, four, large, tailed, fusiform viruses infecting the Sulfolobales have been isolated and characterised. These viruses include the two-tailed bicaudavirus ATV and the three single-tailed monocaudaviruses STSV1, STSV2 and SMV1. At present, little is known about their mechanism of host attachment, DNA injection, DNA packaging and viral release. ATPases have been shown to be involved in some of these processes in eukaryal, bacterial and archaeal viruses indicating that similar ATPase-driven mechanisms are likely to apply to these thermophilic archaeal viruses. All four fusiform virus genomes encode at least one ATPase. There is conservation between ATV p529, ORF526 (STSV1), ORF526 (STSV2), and ORF503 (SMV1) as well as ORF506 of the tadpole-shaped HAV2 (Table 1). However, only ATV p529 has been characterized biochemically. It has a two-domain structure and the domains exhibit ATPase activity as well as DNA-binding and endonuclease activity. The endonuclease activity suggests that p529 may be important in processing the viral genome during encapsidation, where it can be likened to, for example, T4 gp17, which cleaves the viral genome upon headful packaging.

ATV p529 is exceptional in the sense that both its N-terminal ATPase domain and its C-terminal domain bind DNA [40]. DNA binding of the N-terminal domain is negatively influenced by the presence of ATP, suggesting that a conformational change induced by ATP binding inhibits DNA binding. This contrasts with the properties of the phi29 gp16 DNA packaging ATPase, where DNA affinity is induced by a conformational change caused by ATP binding [30]. The isolated C-terminal domain of p529 also exhibits endonuclease activity that is not observed for the wild-type protein, again indicating that activity may be dependent on conformation [40].

Immobilized p529 pulls down the transmembrane oligopeptide-binding host protein SSO1273 considered to be a potential virus receptor [40,91]. Initial DNA ejection into the host could be driven by pressure as described for bacterial, eukaryal and archaeal viruses [87-89,92]. During DNA injection, the double hexameric structure of p529 could provide a pore through which the remaining viral DNA is pulled into the host cell. This would imply that the extra energy needed to finalise viral DNA entry may be provided by the virus independently of host factors. ATV p529 was initially not identified within the virion using SDS-PAGE and Edman degradation of extracted protein bands [69], perhaps due to low abundance. However, a very low concentration of the protein would be sufficient to build a functional complex. Additional studies on p529 are needed to verify its role in viral DNA translocation into the host. Further, it is possible that p529 interacts with SSO1273 on the host’s inner membrane during DNA packaging in STSV2, but this hypothesis has yet to be explored experimentally.

### ATV p618 is a MoxR-type ATPase

ATV p618 was classified as a MoxR-type ATPase [41]. MoxR-type ATPases are found amongst archaea and bacteria, and they have been classified into seven subfamilies [93]. They are proposed to exhibit chaperone-like activities and are involved in various stress response pathways [94]. The role of p618 in the ATV life cycle was investigated by assaying its interactions with nucleic acids and other ATV proteins. ATV p618 is a major virion component [69,70] and it interacts with at least four other virus proteins but not with viral DNA [41]. Three of these proteins p387, p653, and p800 have been found in the virion and p800 can generate long filaments [69,70]. The fourth protein, p892, which carries a von Willebrand factor type A (VWA) domain, interacts strongly with p618 [41]. A homologous protein has been detected in SMV1 virions [44] although not yet in ATV virions. Proteins exhibiting a VWA domain are known to interact with MoxR-like ATPases, as shown for the *E.coli* RavA ATPase [93]. Both p387 and p892 enhance p618 ATPase activity. From this set of observations, it has been concluded that p618 participates in extracellular ATV tail development [41]. This hypothesis was further strengthened by the observation that the fourth interacting ATV protein, p653, is localized in the virion tails [41]. Further studies are needed to localise p618 within the virion tail, and to elucidate its role in tail development.

## Conclusions

Several ATPases are encoded in archaeal viral genomes – both crenarchaeal and euryarchaeal – and these enzymes have been implicated in a variety of functions during the viral life cycle. They include DNA entry into the host, genome packaging into viral capsids, modulation of DNA replication, and as proteases and chaperones, but minimal experimental evidence exists of their precise structures or specific functions. To date, functional studies have only been reported for three ATPases of thermophilic crenarchaeal viruses, and none for the ATPases associated with euryarchaeal viruses. These studies provide a mere glimpse into the functional diversity of these archaeal ATPases. More research is needed in order to answer key questions, such as: i) How does genome packaging occur for the bottle-shaped, droplet-shaped, spindle-shaped and pleomorphic viruses of the archaeal domain? Do these viruses assemble their capsid proteins around the viral genome, or do they utilise an active packaging mechanism involving ATP hydrolysis? ii) To what extent are the functional roles of archaeal viral ATPases inferred from bioinformatical analyses and homology predictions correct? iii) Can the extremophilic viral ATPases yield novel insights into how energy metabolism functions under extreme environmental conditions? iv) Since many archaeal viruses reside in extreme environments, and their virions are stable under extreme conditions, are they generally amenable to important biotechnological and nanotechnological applications? Clearly the field is set for extensive in-depth studies of the diverse ATPases of the archaeal viruses with the results having relevance both for a variety of different viral life cycles and of strong potential interest for biotechnology and nanotechnology.

## Competing interests

The authors declare that they have no competing interest.

## Authors’ contributions

LJH performed the homology modelling of STIV B204, ATV p529 and p618, LJH and SE made the figures, LJH and RAG made the sequence alignments and annotations, LJH, SE, RAG, and SJB wrote the manuscript. All authors read and approved the final manuscript.
